# Supplementary material for: MetaRibo-Seq measures translation in microbiomes
Source: Nat Commun. 2020 Jun 29;11:3268. doi: 10.1038/s41467-020-17081-z (PMC7324362; doi:10.1038/s41467-020-17081-z)
Supplement: Supplementary file 10 — Supplementary Data 7 [file 41467_2020_17081_MOESM10_ESM.zip › File2/Confidence_High_Taxonomy/356745_out.krona.html]

Javascript must be enabled to view this page.

members
magnitude
magnitudeUnassigned
count
unassigned
taxon
rank

356745\_out

147

superkingdom
147
2

976
147
phylum

class
147
200643

order
171549
147

145
1853231
family

283168

SRS011405\_contig\_number\_contig-100\_1216.39319SRS014923\_contig\_number\_contig-100\_7133.95907SRS016267\_contig\_number\_contig-100\_3049.86415SRS017821\_contig\_number\_contig-100\_2513.147327SRS022524\_contig\_number\_3065SRS024625\_contig\_number\_contig-100\_1936.29892SRS045004\_contig\_number\_contig-100\_2712.118949SRS045713\_contig\_number\_9953SRS047014\_contig\_number\_29038SRS048164\_contig\_number\_28043SRS049900\_contig\_number\_15283SRS051031\_contig\_number\_contig-100\_3018.138171SRS051882\_contig\_number\_contig-100\_670.72179SRS055017\_contig\_number\_contig-100\_2698.195530SRS1041039\_contig\_number\_contig-100\_38347.75039SRS1041129\_contig\_number\_contig-100\_7664.7665SRS1054716\_contig\_number\_1698SRS143181\_contig\_number\_contig-100\_15050.59779SRS144603\_contig\_number\_contig-100\_15078.58724
genus
145
19

28118
113
species

SRS011134\_contig\_number\_49514SRS011239\_contig\_number\_19625SRS012849\_contig\_number\_33010SRS012969\_contig\_number\_38214SRS013098\_contig\_number\_20059SRS013476\_contig\_number\_11207SRS013521\_contig\_number\_contig-100\_2850.63639SRS013800\_contig\_number\_22317SRS014412\_contig\_number\_6459SRS015663\_contig\_number\_32507SRS015782\_contig\_number\_32042SRS016095\_contig\_number\_13089SRS016335\_contig\_number\_26450SRS016954\_contig\_number\_contig-100\_2727.128976SRS017247\_contig\_number\_3140SRS017521\_contig\_number\_29831SRS018133\_contig\_number\_11867SRS018623\_contig\_number\_contig-100\_2235.82965SRS018836\_contig\_number\_27087SRS018888\_contig\_number\_contig-100\_862.50418SRS019068\_contig\_number\_62035SRS019161\_contig\_number\_20943SRS019397\_contig\_number\_2757SRS019445\_contig\_number\_20708SRS019601\_contig\_number\_12937SRS019968\_contig\_number\_4414SRS020233\_contig\_number\_contig-100\_880.271153SRS020271\_contig\_number\_contig-100\_811.24575SRS020394\_contig\_number\_contig-100\_429.71253SRS021219\_contig\_number\_contig-100\_150.55116SRS021484\_contig\_number\_16838SRS022609\_contig\_number\_contig-100\_199.213755SRS023526\_contig\_number\_36740SRS023715\_contig\_number\_11693SRS024075\_contig\_number\_9818SRS024132\_contig\_number\_47280SRS024435\_contig\_number\_contig-100\_1139.212332SRS024492\_contig\_number\_11165SRS043001\_contig\_number\_10054SRS043701\_contig\_number\_10898SRS043768\_contig\_number\_29370SRS043841\_contig\_number\_1577SRS045645\_contig\_number\_17867SRS046068\_contig\_number\_45SRS046369\_contig\_number\_9125SRS047044\_contig\_number\_10555SRS047433\_contig\_number\_18470SRS048870\_contig\_number\_16765SRS049446\_contig\_number\_10801SRS049773\_contig\_number\_20591SRS049995\_contig\_number\_2301SRS050026\_contig\_number\_7072SRS050925\_contig\_number\_13875SRS054352\_contig\_number\_15459SRS054905\_contig\_number\_21228SRS056273\_contig\_number\_22325SRS058770\_contig\_number\_14904SRS063040\_contig\_number\_28876SRS063190\_contig\_number\_10678SRS063518\_contig\_number\_14971SRS063985\_contig\_number\_25257SRS064557\_contig\_number\_19140SRS064645\_contig\_number\_contig-100\_1445.1445SRS064757\_contig\_number\_contig-100\_1002.123634SRS075078\_contig\_number\_6981SRS075773\_contig\_number\_1733SRS076756\_contig\_number\_16175SRS077194\_contig\_number\_13202SRS077231\_contig\_number\_contig-100\_43.119737SRS077392\_contig\_number\_27790SRS077641\_contig\_number\_10083SRS077849\_contig\_number\_24726SRS078176\_contig\_number\_15875SRS078242\_contig\_number\_contig-100\_1449.142101SRS078419\_contig\_number\_28878SRS078665\_contig\_number\_3828SRS100021\_contig\_number\_10667SRS101376\_contig\_number\_contig-100\_515.78826SRS103987\_contig\_number\_18767SRS104036\_contig\_number\_contig-100\_451.452SRS1041118\_contig\_number\_contig-100\_2677.82970SRS1041137\_contig\_number\_5812SRS1041145\_contig\_number\_4115SRS104311\_contig\_number\_11289SRS104636\_contig\_number\_17138SRS104975\_contig\_number\_16835SRS105082\_contig\_number\_15741SRS140513\_contig\_number\_12864SRS142503\_contig\_number\_17321SRS142890\_contig\_number\_38045SRS143598\_contig\_number\_13247SRS143876\_contig\_number\_14873SRS144183\_contig\_number\_35587SRS144362\_contig\_number\_24924SRS144506\_contig\_number\_22488SRS145497\_contig\_number\_3985SRS146764\_contig\_number\_18910SRS147139\_contig\_number\_41123SRS147271\_contig\_number\_23805SRS147346\_contig\_number\_35138SRS147557\_contig\_number\_28552SRS148091\_contig\_number\_10584SRS148159\_contig\_number\_36172SRS148196\_contig\_number\_42010SRS148424\_contig\_number\_contig-100\_1367.162912SRS148721\_contig\_number\_33089SRS149181\_contig\_number\_27616SRS149325\_contig\_number\_4878SRS893252\_contig\_number\_18874SRS893279\_contig\_number\_10591SRS893341\_contig\_number\_5248SRS893378\_contig\_number\_21311SRS971275\_contig\_number\_41957

13
1897010

SRS011084\_contig\_number\_25203SRS015431\_contig\_number\_54963SRS015960\_contig\_number\_contig-100\_4109.102523SRS019787\_contig\_number\_contig-100\_3295.35663SRS044535\_contig\_number\_13082SRS046717\_contig\_number\_6949SRS051610\_contig\_number\_10830SRS052697\_contig\_number\_26074SRS065504\_contig\_number\_12256SRS076804\_contig\_number\_19673SRS077589\_contig\_number\_317SRS097958\_contig\_number\_contig-100\_97.98745SRS140492\_contig\_number\_20835
species

family
815
2

816
2
genus

species

SRS053398\_contig\_number\_18231
28116
1

1
310297
species

SRS017433\_contig\_number\_4871
